# Supplementary figures and images for: Subcellular proteomics of the protist Paradiplonema papillatum reveals the digestive capacity of the cell membrane and the plasticity of peroxisomes across euglenozoans
Source: PLoS Biol. 2025 Dec 3;23(12):e3003319. doi: 10.1371/journal.pbio.3003319 (PMC12697944; doi:10.1371/journal.pbio.3003319)

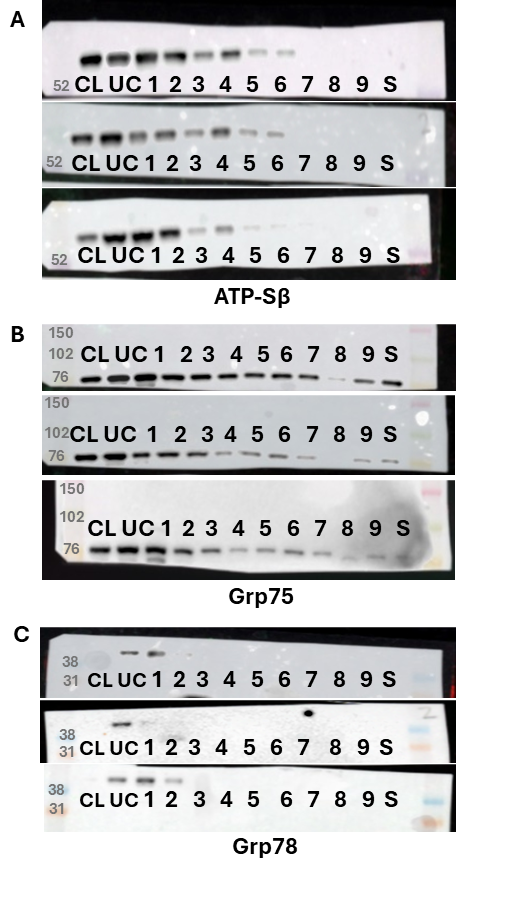

Supplement: S1 Fig — 10 μg of protein has been loaded for each fraction generated via differential centrifugation in addition to the initial cell lysate (CL). ATP synthase-β antibody used at 1:10,000 ratio (A), Grp75 antibody used in 1:1,000 (B), and Grp78 (C) which displays non-specific signal. Unlysed cells (UC), Supernatant (S). Marker band molecular weights (kDa) indicated in dark gray on the leftmost lane of blots. (TIF) [file pbio.3003319.s001.tif]

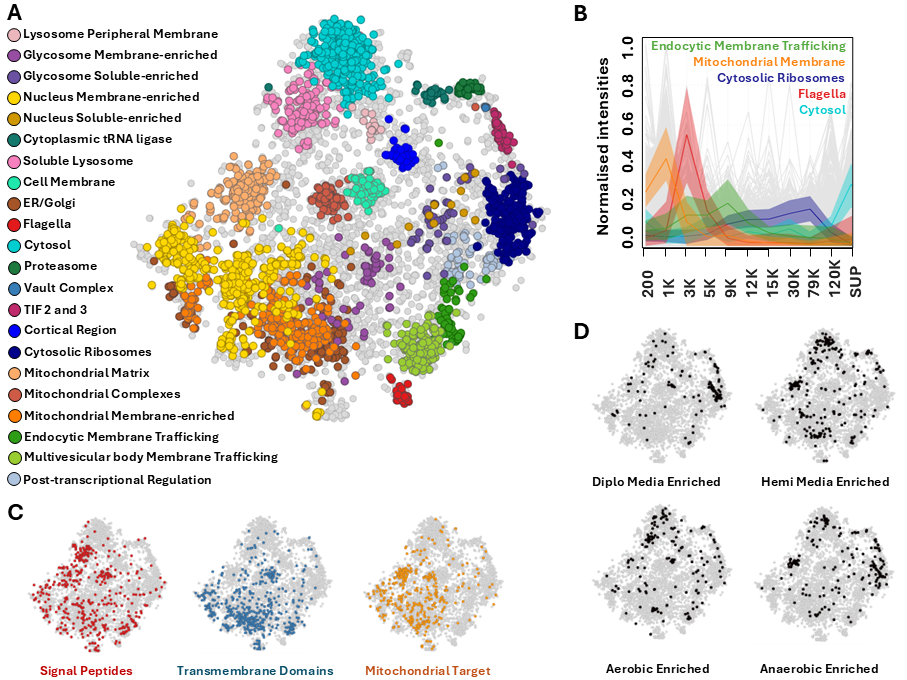

Supplement: S2 Fig — (A) Full dataset displaying clustered predictions displayed for 4,780 proteins across 22 cell compartments. Predictions were generated via support vector modeling conducted on fractional profiles of marker proteins, applied to the remaining dataset. (B) Selected fractional abundances of marker proteins across one replicate of this experiment, representing distinct profiles that facilitate predictive clustering (SUP, Supernatant). (C) Software prediction for protein features of signal peptides, transmembrane domains, and mitochondrial target peptides across dataset, demonstrating accumulation across certain defined compartments. (D) Proteins determined to be enriched in varying nutrient media (Diplo or Hemi) or cultivation conditions (aerobic or anaerobic) from a conditional study of Paradiplonema papillatum [8]. Additional information for all proteins available in Tables B, C and D in S1 Data. (TIF) [file pbio.3003319.s002.tif]

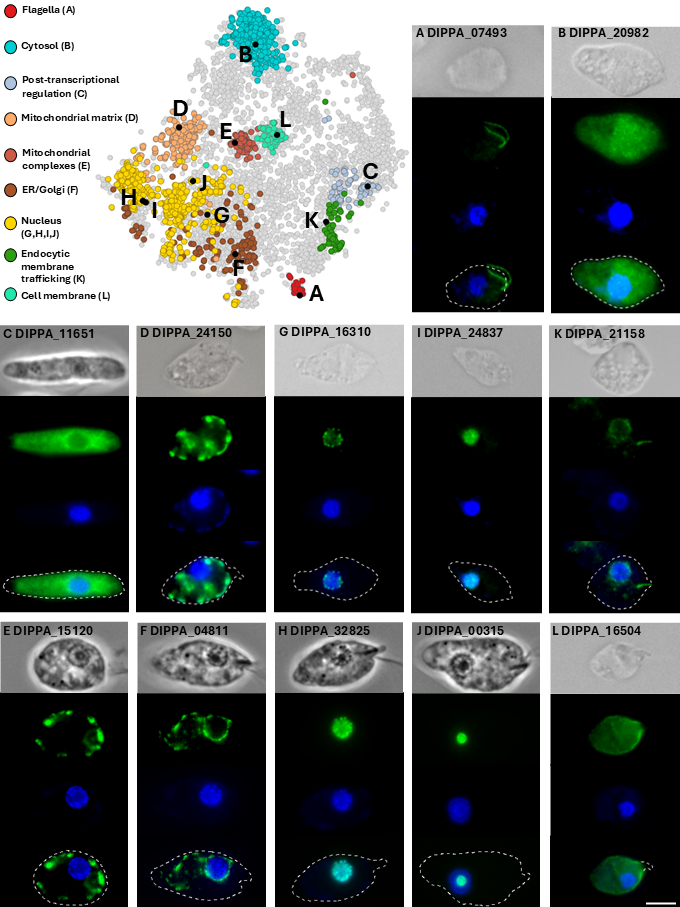

Supplement: S3 Fig — Tagged proteins highlighted (black) among relevant predicted clusters, resolved on neighbor-averaged and zero imputed t-SNE. Individual cell lines were generated via endogenous tagging and imaged through fluorescence microscopy for comparison with compartment relevant protein was predicted to. In descending order, panels depict phase contrast, epitope signal (green), nuclear and mitochondrial DNA (blue), with merges below additionally displaying cell membrane outlines traces for all images, excepting L, which shows only trace of the papilla, which lacks epitope signal. All imaged cells are oriented with their apical regions facing right and posterior facing left. Scale bar represents 5 μm. Further information on cell lines is available in Table E in S1 Data. (TIF) [file pbio.3003319.s003.tif]

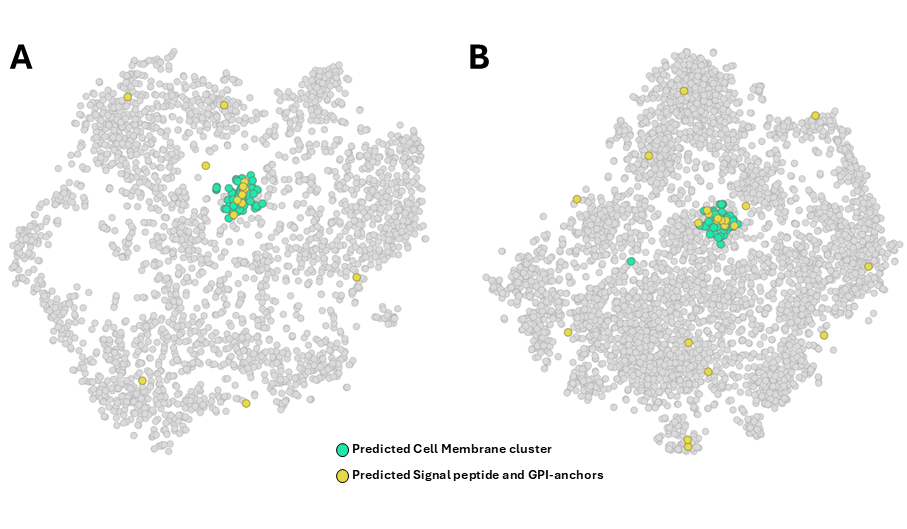

Supplement: S4 Fig — t-SNE imputed via neighbor-averaging (A) as well as zeroed dataset (B). Signal peptides predicted via Signal P 6.0 with a confidence threshold greater than 0.9, in tandem with NetGPI 1.1 used for GPI predictions. Further information is available in Table D in S1 Data. (TIF) [file pbio.3003319.s004.tif]

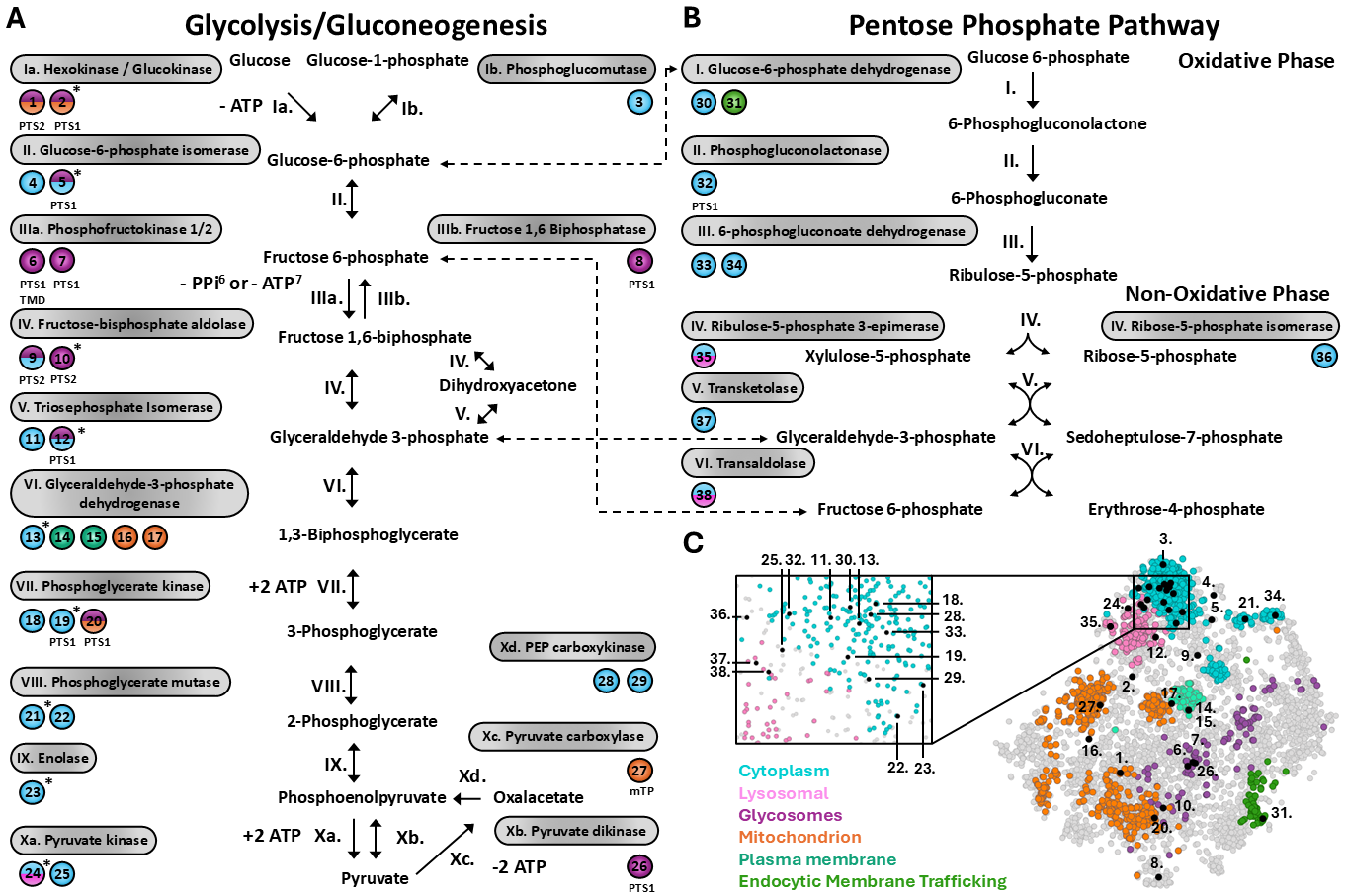

Supplement: S5 Fig — Localization of relevant enzymes across glycolysis/gluconeogenesis (A) and pentose phosphate pathway (B), resolved on neighbor-average and zero imputed t-SNE (C) with relevant localization clusters highlighted. Peroxisomal target sequences (PTS), mitochondrial target peptides (mTP), and transmembrane domains (TMD) are indicated. Proteins previously localized via anti-sera immunolocalizations indicated with *, metabolite shunts between two pathways indicated with dotted arrows. Split coloring of proteins represents their manual designations to the cytosol (24,25,38) or indicates the possibility of glycosomal dual localizations between the cytosol and glycosomes (1,2,5,9,12,20), based on inspection of fractionation profiles (S6 Fig) and targeting signals. Further information is available in Table G in S1 Data. (TIF) [file pbio.3003319.s005.tif]

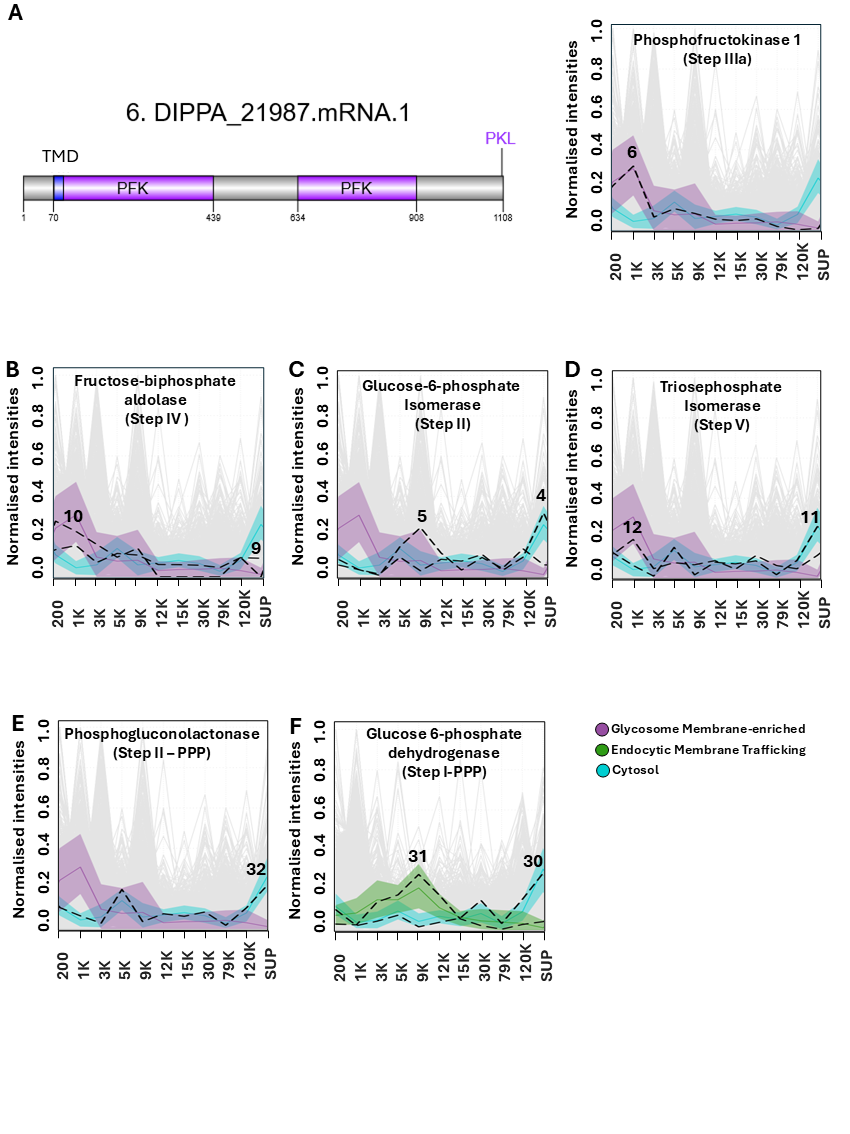

Supplement: S6 Fig — Schematic depiction of DIPPA_21987, phosphofructokinase 1 showing phosphofructokinase (PFK) domains, transmembrane domain (TMD), and Peroxisomal Target Signal along with fractional analysis (A), along with fractional profiles of relevant enzymes across glycolysis/gluconeogenesis (B) compared to marker proteins of the cytosol, glycosomes, and endocytic membrane trafficking markers. (TIF) [file pbio.3003319.s006.tif]

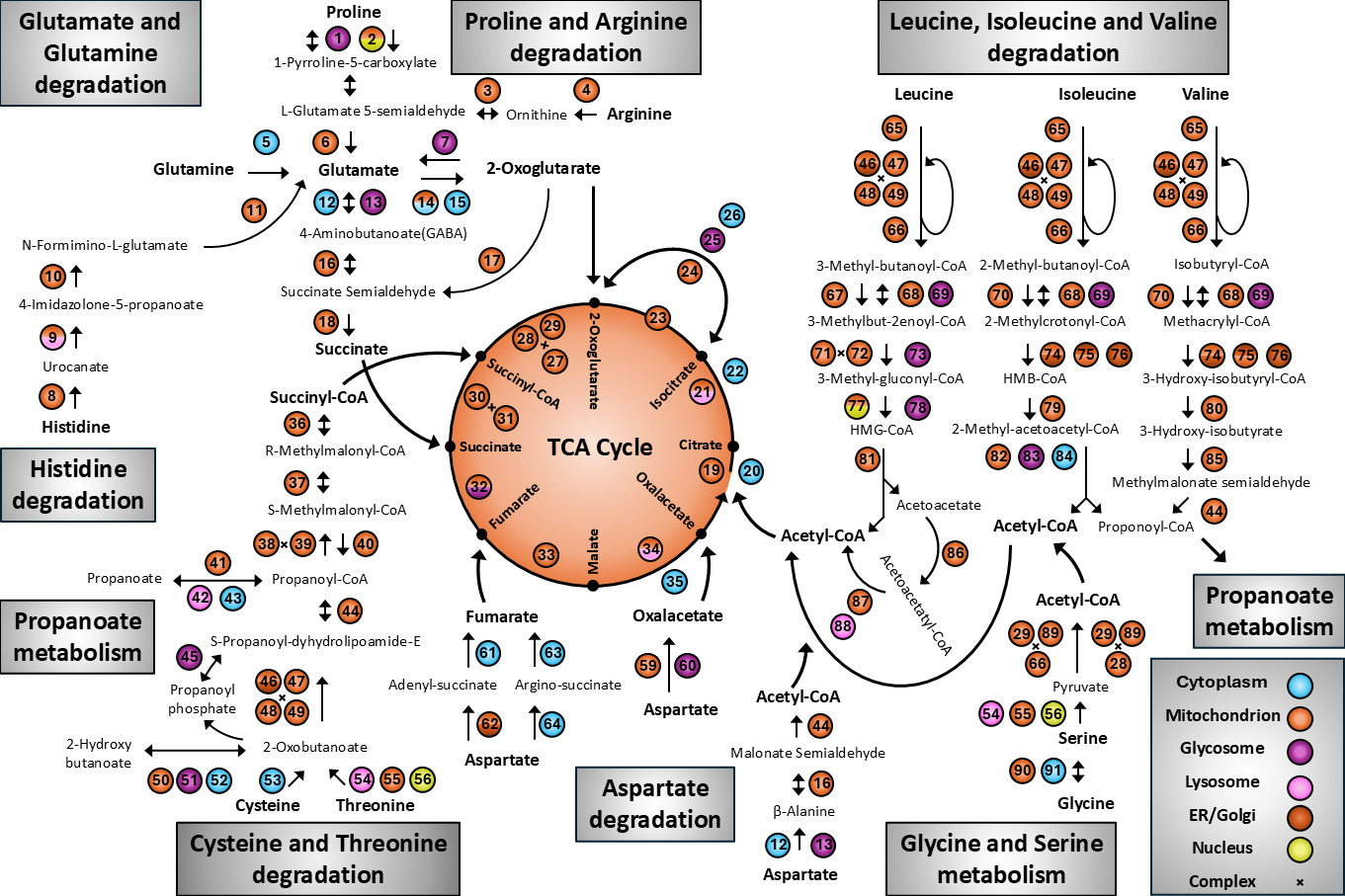

Supplement: S7 Fig — AAs and metabolites of the TCA cycle are indicated in bold. Propanoate metabolism, which involves intermediates of certain AA digestion, is also depicted. Split coloring indicates manual annotation for specific enzymes based on certain target peptides or candidate function, on top, versus contrasting predictions below (e.g., Enzyme 2: proline dehydrogenase, we designate to the mitochondrion, despite low confidence predictions to the nucleus). Further information is available in Table H in S1 Data. (TIF) [file pbio.3003319.s007.tif]
