# Supplementary material for: Subcellular proteomics of the protist Paradiplonema papillatum reveals the digestive capacity of the cell membrane and the plasticity of peroxisomes across euglenozoans
Source: PLoS Biol. 2025 Dec 3;23(12):e3003319. doi: 10.1371/journal.pbio.3003319 (PMC12697944; doi:10.1371/journal.pbio.3003319)
Supplement: S1 Raw Images — (PDF) [file pbio.3003319.s010.pdf]

ATP-Sβ

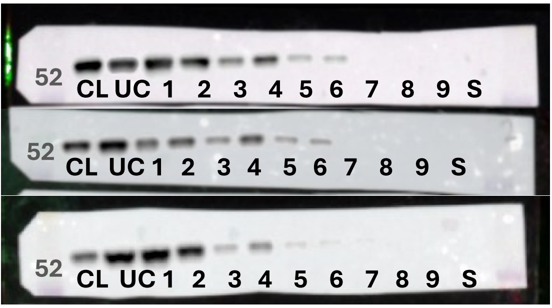

Replicate 1

Replicate 2

Replicate 3

Grp75

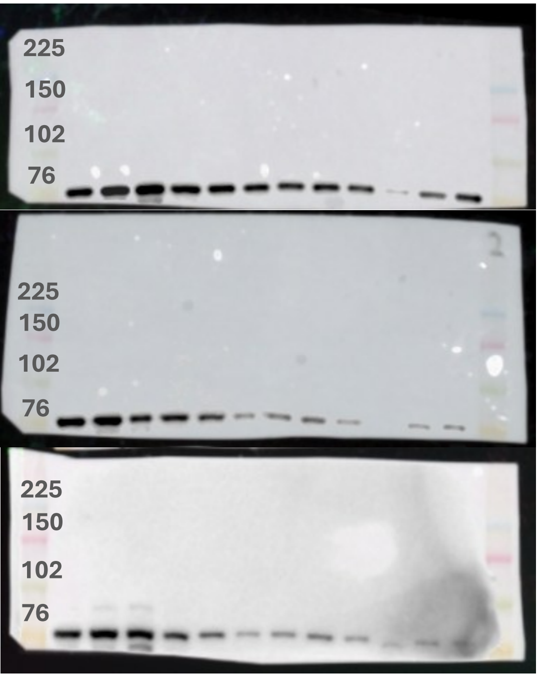

Replicate 1

Replicate 2

Replicate 3

Grp78

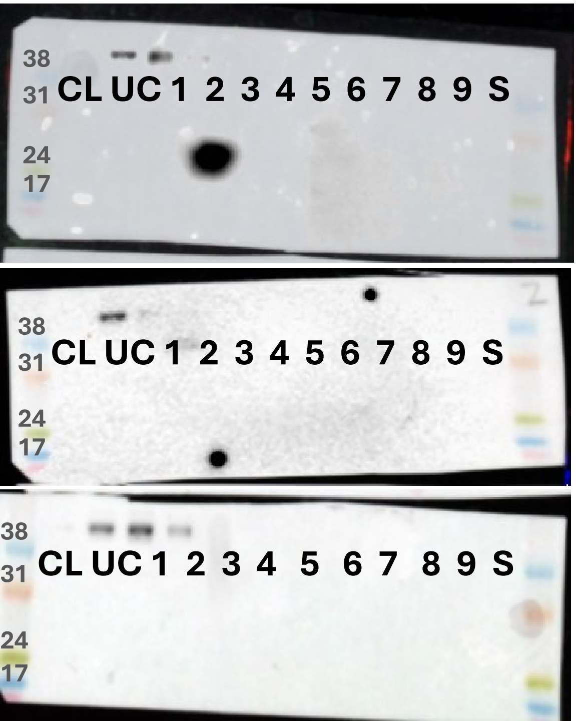

Replicate 1

Replicate 2

Replicate 3
