# Supplementary material for: Subcellular proteomics of the protist Paradiplonema papillatum reveals the digestive capacity of the cell membrane and the plasticity of peroxisomes across euglenozoans
Source: PLoS Biol. 2025 Dec 3;23(12):e3003319. doi: 10.1371/journal.pbio.3003319 (PMC12697944; doi:10.1371/journal.pbio.3003319)
Supplement: Attachment — Submitted filename: Hammond Response to Reviewers_Final.docx [file pbio.3003319.s013.docx]

Response to editor and reviewer comments:

Author responses are in **BOLD**

Editor comments:

Based on the reviews, we are likely to accept this manuscript for publication, provided you satisfactorily address the points raised by the reviewers. However, we would like you to resubmit the manuscript as a Methods and Resources manuscript, thus please select that type of article from the drop-down menu when you resubmit a revised version. Please also make sure to address the data and other policy-related requests stated below my signature.

**Thank you for the opportunity to revise our manuscript. Based on these requests, we have resubmitted the manuscript as a “Methods and Resource”, we have also changed the manuscript title:**

“**Subcellular proteomics of the protist *Paradiplonema papillatum* reveals the digestive capacity of the cell membrane and the plasticity of peroxisomes across euglenozoans”**

Note that we do not require all raw data. Rather, we ask that all individual quantitative observations that underlie the data summarized in the figures and results of your paper be made available in one of the following forms:

1) Supplementary files (e.g., excel). Please ensure that all data files are uploaded as 'Supporting Information' and are invariably referred to (in the manuscript, figure legends, and the Description field when uploading your files) using the following format verbatim: S1 Data, S2 Data, etc. Multiple panels of a single or even several figures can be included as multiple sheets in one excel file that is saved using exactly the following convention: S1_Data.xlsx (using an underscore).

**We have formatted our Supplementary files as S1_Data, S2_Data in file name, and refer to them as such in the manuscript.**

2) Deposition in a publicly available repository. Please also provide the accession code or a reviewer link so that we may view your data before publication.

**Pride Dataset PXD065121 can be accessed for review as,**

**Username: reviewer_pxd065121@ebi.ac.uk**

**Password: jDgYK6ALGDu8**

Regardless of the method selected, please ensure that you provide the individual numerical values that underlie the summary data displayed in the following figure panels as they are essential for readers to assess your analysis and to reproduce it:

Fig. 1B; Fig. S2B and Fig. 6A-F

**This information can now be found in Table S1 of S1_Data file.**

Reviewer 1:

The authors performed sub-cellular fractionation via differential centrifugation followed by proteomics on P. papillatum to identify proteins enriched in different cellular compartments. They did a seemingly thorough job, tested multiple medias and growth conditions and followed up on a few different groups of proteins of interest in their dataset. After manual annotation of 368 proteins and prediction of sub cellular localization for 2,435 they then tested their predictions and confirmed their accuracy by native protein tagging. From this analysis they also identified some interesting protein localizations that will likely fuel a lot of future research and be of interest to the broader community.

The set of CASzymes showed an enrichment of signal-peptide containing proteins, leading the authors to hypothesize a mechanism of extracellular digestion and uptake of nutrients via the cytopharynx. Additionally, they make other predictions about CASzymes and sugar transporters function in various cellular compartments. They also interpret that these CASzymes are constitutively expressed under all of the conditions tested which supports the hypothesis that plants and algae are a primary food source of P. papillatum. Next the authors explored a set of proteins related to the glycolytic pathway and the pentose phosphate pathway. Most of their findings seem to agree with previous findings and/or support existing hypotheses. They did identify some proteins that were localized to compartments that had not been previously described.

Overall their findings shed new light on the evolutionary similarities and differences in comparison to kinetoplastids and reveal a trove of information about the metabolic pathways in a model diplonemid.

As stated above, this work provides independent support for many existing hypothesis as well as providing new predictions about some of the metabolic pathways that exist in diplonemids. The work presented here seems highly impactful to the field as well as the broader community. This work also appears to be technically sound. I would recommend this work for publication in PLoS Biology.

**We thank the reviewer for the positive feedback and their acknowledgement of the value of our work.**

Most of the language throughout the paper is careful to discuss predictions, but there is at least one place where the language veers more toward demonstration of function, which should be avoided in the absence of functional biochemical studies. (Line 331).

**We have modified the wording concerning the potential enzymatic capacity to digest certain propanoate metabolites (Line 342 with tracked changes), rather than stating this as a foregone conclusion.**

**“*We additionally reveal the possibility of diplonemids to process the fatty acid propanoate to propanoyl-CoA, which would allow incorporation into AA intermediate-processing pathways within the mitochondrion, representing a functional distinction from kinetoplastids*”**

Reviewer 2:

I really don't think the CAZymes you mention throughout is the correct term. These nearly all appear to be **Glycoside Hydrolases (GHs)** and not including glycosyl transferases, auxiliary activities or binding molecules (CBMS) which are also CAZymes. This a bit implies there could be extensive carbohydrate synthesis on the surface if there were lots of GTs. I would prefer this be changed to Glycoside Hydrolases (GH) throughout. Possibly **carbohydrate degrading** **enzymes**.

**We elected to refer to these enzymes as carbohydrate degrading enzymes (CDZymes) as suggested by reviewer two, while most of these enzymes are glycoside hydrolases, a few that we draw attention to are not, so this is probably the best abbreviation to use.**

Figure 3A implies there are multiple enzyme domains in eg DIPPA17995 - are these all intact whole enzymes stitched together? Or parts of the active domain split up with spacers between? Were there any carbohydrate binding modules on any of these? Or protein-protein interaction domains to make something akin to a cellulosome? Could the authors make some comment as to this as it is unusual enzyme topology.

**Here, we have analysed proteins of interest with multiple complete enzyme domains, detailing their likely origin (partial gene duplication) and their potential function of these repeat regions (tertiary structure formation).**

**Lines 197-202:**

**“We note that several proteins of pectin degradation (DIPPA_26123, DIPPA_17995, DIPPA_19114) possess multiple complete enzymatic domains of the same category following their signal peptide (Fig. 3A). Internal repeat regions of high similarity in both proteins likely arose from genetic duplication across these regions, with the further presence of repeated parallel beta helix regions within these enzyme domains suggesting a coiled tertiary structure for both of these extracellular-exposed, embedded proteins.”**

Not sure this shows P. papillatum "preys on plants and algae" (line 350), as they could be scavenging shed cell wall or dead cells. Please remove reference to predator/prey and just say feeds upon.

**In response, we have replaced all references to *P. papillatum* as “preying” on plants and algae with feeding (Lines 81,242 and 361 with tracked changes).**

Line 116: "underwent and imputation" Is this meant to be "underwent imputation" or is it "underwent XXX and imputation"?

**Thank you for noticing this typo. In line 119 (tracked changes), we have removed the additional ‘and’.**
